# Genomic Characterization of a Multidrug-Resistant Aeromonas caviae Isolate Carrying a Novel *bla*_KPC-2_-Harbouring Plasmid and an IMP-4-Encoding Phage-like Plasmid

**DOI:** 10.1128/spectrum.00840-22

**Published:** 2022-07-13

**Authors:** Ying Li, Yichuan Qiu, Chengju Fang, Xiaoyi Dai, Luhua Zhang

**Affiliations:** a The School of Basic Medical Science and Public Center of Experimental Technology, Southwest Medical University, Luzhou, Sichuan Province, China; University of L'Aquila

**Keywords:** *Aeromonas caviae*, blaIMP-4, blaKPC-2, carbapenemase, phage-like plasmid

## LETTER

Carbapenem resistance, mainly mediated by the production of carbapenemases, poses a serious threat to global public health (1). KPC-2 and IMP-4 serve as two important representatives of carbapenemases that have been commonly found on transmissible plasmids in various bacterial species (2–4). There were limited reports of the co-production of KPC-2 and IMP-4, only in clinical Klebsiella pneumoniae and Klebsiella oxytoca isolates in China (5–8). Here, we characterize a multidrug-resistant (MDR) Aeromonas caviae isolate in China, and report for the first time the simultaneous presence of *bla*_KPC-2_ and *bla*_IMP-4_, carried by two new types of plasmids in this species.

A. caviae strain SCLZS52 was isolated from the influx mainstream of the wastewater treatment plant of the affiliated hospital of Southwest Medical University, in August 2019, in Sichuan, China. Antimicrobial susceptibility testing was performed using the broth microdilution method and was interpreted according to Clinical and Laboratory Standards Institute documents M45 (9). SCLZS52 was resistant to meropenem, cefotaxime, cefoxitin, ciprofloxacin, and gentamicin, intermediate to tetracycline, and susceptible to tigecycline, amikacin, and chloramphenicol. It was subjected to whole genome sequencing (WGS) by using both the MinION and Illumina HiSeq 2000 sequencers. The assembly and bioinformatic analyses of the genome were performed as previously described (10). WGS data revealed that the SCLZS52 belongs to A. caviae, and it is comprised of a 4,718,963-bp circular chromosome and eight plasmids ranging from 4,076 bp to 113,450 bp in size (Table S1 in the supplemental material). SCLZS52 has 27 known acquired antimicrobial resistance genes (ARGs) mediating multidrug resistance, including two carbapenemase-encoding genes *bla*_KPC-2_ and *bla*_IMP-4_ located on two different plasmids (Table S1). Conjugation experiments were carried out using Escherichia coli strains J53 and EC600 as recipients (10, 11). However, no transconjugant was obtained after repeated attempts, suggesting that both carbapenemase determinants were not transferable, which was consistent with that no conjugative elements were detected on the plasmids.

**FIG 1:**
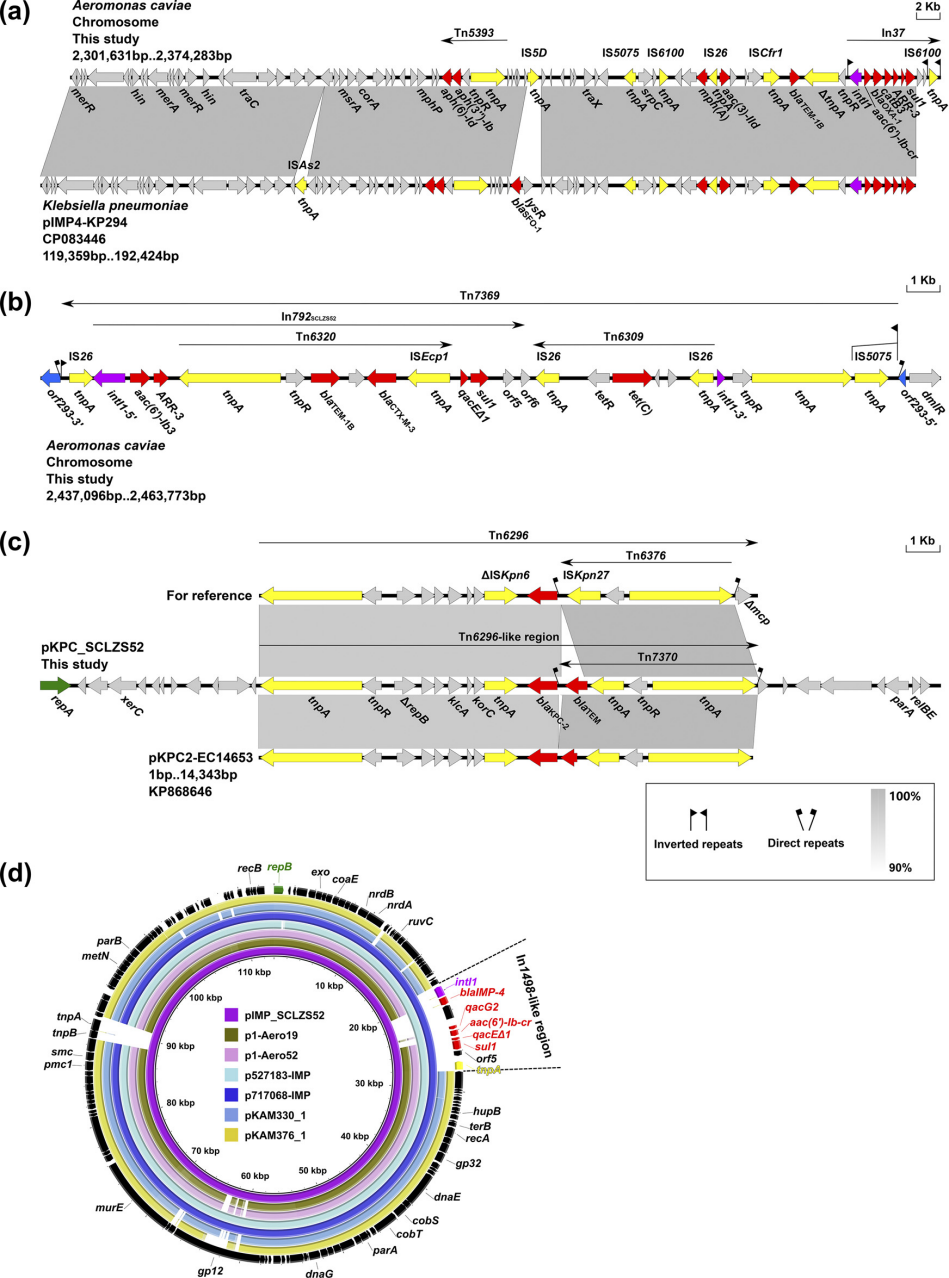
Genetic features of SCLZS52. (a) Comparison of the chromosomal MDR-1 of SCLZS52 with the corresponding region on plasmid pIMP4-KP294. Genes are denoted by arrows. Resistance genes, mobile genetic elements and *intI1* are indicated in red, yellow, and purple, respectively. Regions of >90% homology are indicated by gray shadings. Δ represents truncated genes or mobile genetic elements. (b) The configuration of chromosomal MDR-2 region of SCLZS52. The interrupted gene *orf293* is marked in blue. (c) Organization of the plasmid pKPC_SCLZS52 and comparison to related regions. The *repA* is indicated in green. (d) Comparison of pIMP_SCLZS52 with similar plasmids. pIMP_SCLZS52 was used as the reference. Dark arrows at the outer circles show open reading frames from pIMP_SCLZS52, with *repB* marked in green. Resistance genes, mobile genetic elements and *intI1* in the accessory module are highlighted by colored arrows. Gaps indicate regions that were missing in the respective plasmid compared to pIMP_SCLZS52. Plasmids from inside to outside are pIMP_SCLZS52 (CP091177), p1_Aero19 (CP068233), p1_Aero52 (CP066814), p527183-IMP (MN961666), p717068-IMP (MN629346), pKAM330_1 (AP023399), and pKAM376_1(AP024403).

Twenty ARGs were located on the chromosome of SCLZS52, which are mainly clustered in two MDR regions, designated MDR-1 and MDR-2 (Fig. S1 in the supplemental material; Fig. 1a and b). The 41-kb MDR-1 shows >99.9% identity at 98% coverage to that of plasmids from Klebsiella, such as pIMP4-KP294 (CP083446, patient, China, 2020) and pKP1814-1 (KX839207, patient, China, 2011), suggesting that SCLZS52 may capture this segment from Klebsiella plasmids, most likely by homologous recombination (Fig. 1a). The 24-kb MDR-2 is sequentially organized as an In*792* with an IS*26*-mediated interruption at *intI1* and an insertion of Tn*6320* downstream of its gene cassette, an intact Tn*6309*, and a core transposition module *tnpAR-res* with an IS*5075*-disrupted IRL (inverted repeat left) (Fig. 1b). The complex chimera structure is further identified as a novel transposon designated Tn*7369* by the Transposon Registry. Tn*7369* splits *orf293* into two separate parts, leaving 6-bp direct repeats (DRs; target site duplication signals for transposition, TTCATA). BLASTn analysis revealed that Tn*7369* is not common outside of SCLZS52 and its prevalence remains unclear.

The plasmid pKPC_SCLZS52 (CP091179) is 26,128 bp in size, carries *bla*_TEM_ in addition to *bla*_KPC-2_, and could not be assigned into any known incompatibility group. The deduced replication protein RepA belongs to the PriCT_1 superfamily (PF08708), and matches RepA proteins of two *Aeromonas* plasmids (WP_171281265 and WP_139750798) with >96.66% amino acid identity at >99% coverage. The backbone of pKPC_SCLZS52 had only 30% coverage (76.13% nucleotide identity) to its closest match plasmid unnamed2 (CP083946) from Aeromonas hydrophila, indicating that pKPC_SCLZS52 is a novel type of plasmid carrying *bla*_KPC-2_. In pKPC_SCLZS52, *bla*_KPC-2_ is contained in a Tn*6296*-like structure, wherein a novel transposon designated Tn*7370*, instead of Tn*6376* in Tn*6296*, is located upstream of *bla*_KPC-2_, and the terminal Δ*mcp* is deleted (Fig. 1c). Tn*7370* is a Tn*3*-derived transposon with an insertion of IS*Kpn27* upstream of *bla*_TEM_. By BLASTn, the closest match of the *bla*_KPC-2_ region of pKPC_SCLZS52 is that of plasmid pKPC2-EC14653 (98% coverage, 98.27% identity) from Enterobacter cloacae (KP868646, patient, China, 2014), except for a 127-bp deletion between *bla*_KPC-2_ and *bla*_TEM_ in the latter case, indicating a common origin of them.

The plasmid pIMP_SCLZS52 (CP091177) is 113,450 bp, wherein *bla*_IMP-4_ is contained in an In*1498*-like class I integron, which differed from In*1498* mainly by insertion of a *ltrA* (encoding a putative retron-type RNA-directed DNA polymerase) downstream of *bla*_IMP-4_ and an IS*6100* of the 3′-CS (3′ conserved segment). pIMP_SCLZS52 encodes a replication protein RepB of the Rep_3 superfamily (pfam10134) that does not belong to any known incompatibility group. Outside of the replication module, a cluster of genes encoding putative phage proteins are scattered in the remaining 112.1-kb region of the pIMP_SCLZS52 (Table S2 in the supplemental material). Of them, 39 genes are homologous to those of the Pseudomonas phage nickie (MG018927, wastewater, Denmark). The complete sequences of pIMP_SCLZS52 match six *Aeromonas* plasmids from humans and the environment with >96.53% nucleotide identity at 86–97% coverage (Fig. 1d), which constitutes a novel group of plasmids comprising a relatively conserved backbone and an accessory module carrying different ARGs, including *bla*_IMP-4_ (Fig. 1d, Fig. S2).

In conclusion, this study characterized the genomic features of an MDR *A. caviae* isolate, which harbors a novel type of plasmid carrying *bla*_KPC-2_ and a phage-like plasmid carrying *bla*_IMP-4_. Our work may shed new insights into the high plasticity of mobile genetic elements as vehicles in mediating the dissemination of ARGs.

### Data availability.

Complete sequences of the chromosome and plasmids of SCLZS52 were deposited in GenBank under accession numbers CP091176-CP091184.
