# Supplementary material for: Genomic Characterization of a Multidrug-Resistant Aeromonas caviae Isolate Carrying a Novel blaKPC-2-Harbouring Plasmid and an IMP-4-Encoding Phage-like Plasmid
Source: Microbiol Spectr. 2022 Jul 13;10(4):e00840-22. doi: 10.1128/spectrum.00840-22 (PMC9430807; doi:10.1128/spectrum.00840-22)
Supplement: Supplemental file 1 — Supplemental material. Download spectrum.00840-22-s0001.pdf, PDF file, 1.3 MB [file spectrum.00840-22-s0001.pdf]

Table S1 Summary of the genetic features of *Aeromonas caviae* SCLZS52.

Chromosome /plasmid	Accession no.	Length (bp)	GC%	No. of predicted ORFs	Replicon type	Drug resistance gene
Chromosome	CP091176	4,718,963	61.46	4,518	–	*aph(3'')-Ib*, *aac(3)-IId*, *aac(6')-Ib-cr*[a], *aac(6')-Ib3*, *aph(6)-Id*, *tet(C)*, *mph(A)*, *sul1*[a], *ARR-3*[a], *bla*$_{CTX-M-3}$, *bla*$_{TEM}$, *bla*$_{TEM-1B}$, *bla*$_{MOX-5}$, *bla*$_{OXA-1}$, *qacE*[a], *catB3*
pIMP_SCLZS52	CP091177	113,450	58.45	132	UT	*bla*$_{IMP-4}$, *aac(6')-Ib-cr*, *qacE*, *sul1*
pKPC_SCLZS52	CP091179	26,128	56.78	25	UT	*bla*$_{KPC-2}$, *bla*$_{TEM}$
pqnrS2_SCLZS52	CP091180	9,639	56.99	9	IncQ2	*qnrS2*
p3_SCLZS52	CP091178	44,851	58.34	56	UT	
p5_SCLZS52	CP091181	7,196	55.57	11	UT	
p6_SCLZS52	CP091182	7,070	46.69	11	UT	
p7_SCLZS52	CP091183	5,515	56.65	8	UT	
p8_SCLZS52	CP091184	4,076	50.52	5	UT	

–, not available; UT, untypable.

Table S2 Features of pIMP_SCLZS52.

Feature	Position	Function
001	1..927	Replication initiator protein of Rep_3 superfamily
002	complement (1277..1610)	Hypothetical protein
003	complement (1861..2124)	Hypothetical protein
004	complement (2182..3234)	Ribonuclease E
005	complement (3375..4070)	Haloacid dehalogenase-like hydrolase
006	complement (4094..4696)	Putative exonuclease
007	complement (4693..5124)	Hypothetical protein
008	complement (5124..5720)	Hypothetical protein
009	complement (5808..6404)	Dephospho-CoA kinase
010	complement (6404..7087)	Hypothetical protein
011	complement (7091..7579)	Dihydrofolate reductase type 1
012	complement (7576..8412)	Thymidylate synthase
013	complement (8558..9526)	Ribonucleotide-diphosphate reductase subunit beta
014	complement (9537..11264)	Ribonucleotide-diphosphate reductase subunit alpha
015	complement (11583..11930)	Hypothetical protein
016	complement (12030..12221)	Hypothetical protein
017	complement (12367..12645)	Hypothetical protein
018	complement (12642..12788)	Hypothetical protein
019	complement (12796..13374)	Putative ribonuclease H-like domain-containing protein
020	complement (13400..13918)	Hypothetical protein
021	complement (13956..15917)	Putative exonuclease subunit 2
022	complement (15914..16165)	Hypothetical protein
023	complement (16162..16596)	Hypothetical protein
024	complement (16593..16871)	Hypothetical protein
025	complement (16868..17929)	Putative exonuclease subunit 1
026	complement (17926..18174)	Hypothetical protein
027	18482..18658	Hypothetical protein
028	complement (18672..19169)	Hypothetical protein
029	complement (19439..20452)	Integrase IntI1
030	20605..21345	Beta-lactamase IMP-4
031	complement (21435..22784)	Tail assembly protein
032	23524..23856	Putative quaternary ammonium compound-resistance protein
033	23983..24537	Aminoglycoside N(6')-acetyltransferase
034	24706..24987	Putative quaternary ammonium compound-resistance protein
035	25020..25886	Dihydropteroate synthase

036	26014..26514	Puromycin N-acetyltransferase
037	complement (27043..27837)	Transposase
038	complement (28001..29686)	Hypothetical protein
039	complement (29694..29936)	Hypothetical protein
040	complement (29938..30171)	Hypothetical protein
041	complement (30240..30389)	Hypothetical protein
042	complement (30450..30851)	Hypothetical protein
043	complement (30903..31142)	TraG-like protein
044	complement (31194..31442)	Hypothetical protein
045	complement (31497..31883)	Hypothetical protein
046	complement (31876..32151)	DNA-binding protein HU-beta
047	complement (32226..32711)	Hypothetical protein
048	complement (32827..32988)	Hypothetical protein
049	complement (32985..33431)	Putative single-stranded DNA binding protein
050	complement (33534..34553)	Putative RecA-like recombinase
051	complement (34573..35169)	Putative DNA methyltransferase
052	complement (35172..36104)	DNA polymerase I
053	complement (36160..37125)	Gp32
054	complement (37291..37851)	Hypothetical protein
055	complement (37862..38143)	Hypothetical protein
056	complement (38167..38562)	Hypothetical protein
057	complement (38637..41978)	DNA polymerase III alpha subunit
058	complement (41965..42567)	Hypothetical protein
059	complement (42564..43760)	Putative porphyrin biosynthetic protein
060	complement (43885..46203)	Putative porphyrin biosynthetic protein
061	complement (46285..46740)	DNA adenine methyltransferase
062	complement (46886..47137)	Hypothetical protein
063	complement (47134..47895)	ParA-like protein
064	complement (47892..48530)	Hypothetical protein
065	complement (48660..50060)	Putative DNA ligase
066	complement (50057..50776)	Hypothetical protein
067	complement (50814..51392)	Hypothetical protein
068	complement (51407..52108)	Hypothetical protein
069	complement (52170..53276)	Putative DNA primase
070	complement (53318..53434)	Hypothetical protein
071	complement (53427..53789)	Holin
072	complement (53801..55165)	Putative DNA helicase

073	complement (55174..55497)	Hypothetical protein
074	complement (55619..55975)	Hypothetical protein
075	complement (56035..56790)	Hypothetical protein
076	complement (56972..57250)	Hypothetical protein
077	complement (57343..57588)	Primase
078	complement (57597..58088)	Hypothetical protein
079	complement (58072..66867)	Phage tail fiber protein
080	complement (66945..67541)	Hypothetical protein
081	complement (67541..67849)	Hypothetical protein
082	complement (67849..75963)	2, 6-diaminopimelate ligase
083	complement (76330..76956)	Hypothetical protein
084	complement (76989..77357)	Hypothetical protein
085	complement (77357..80968)	Putative tail tape measure protein
086	complement (80980..81285)	Hypothetical protein
087	complement (81294..81614)	Hypothetical protein
088	complement (81701..82228)	Putative Ig-like domain-containing protein
089	complement (82239..82619)	Hypothetical protein
090	complement (82619..83152)	Hypothetical protein
091	complement (83139..83486)	Hypothetical protein
092	complement (83489..84190)	Hypothetical protein
093	complement (84183..84962)	Hypothetical protein
094	complement (84968..85438)	Hypothetical protein
095	complement (85492..86391)	Putative major capsid protein
096	complement (86463..87293)	Hypothetical protein
097	complement (87342..88637)	Hypothetical protein
098	complement (88685..89425)	Mobile element protein
099	complement (89412..90572)	Transposase
100	complement (90844..91293)	Hypothetical protein
101	complement (91350..92588)	Putative terminase large subunit
102	complement (92588..93172)	Putative DNA-binding protein
103	complement (93819..94100)	Hypothetical protein
104	complement (94091..94966)	Putative ABC transporter ATP-binding protein
105	complement (94963..95412)	Structural protein
106	complement (95409..95876)	Hypothetical protein
107	complement (95876..96550)	Putative ABC transporter ATP-binding protein
108	complement (96519..97016)	Hypothetical protein
109	complement (97009..97671)	Putative ParB-like nuclease domain-containing protein

110	complement (97671..98423)	Putative ParB-like nuclease domain-containing protein
111	98526..100217	Putative helicase
112	100214..100513	Hypothetical protein
113	100510..100986	Hypothetical protein
114	101021..101470	Putative baseplate hub subunit and tail lysozyme
115	101480..101701	Hypothetical protein
116	102258..102695	Hypothetical protein
117	102695..102853	Hypothetical protein
118	102938..103216	Hypothetical protein
119	103487..104113	Hypothetical protein
120	104437..104574	Hypothetical protein
121	complement (104587..105396)	Hypothetical protein
122	105656..105874	Hypothetical protein
123	complement (106147..106374)	Hypothetical protein
124	complement (106430..107128)	Biotin/lipoate A/B protein ligase
125	complement (108490..108801)	Hypothetical protein
126	complement (108801..109088)	Hypothetical protein
127	complement (109133..109456)	Hypothetical protein
128	complement (109850..110293)	Hypothetical protein
129	complement (110666..111112)	Hypothetical protein
130	complement (111116..111361)	Hypothetical protein
131	complement (111358..111705)	Hypothetical protein
132	complement (111738..112514)	Hypothetical protein

4

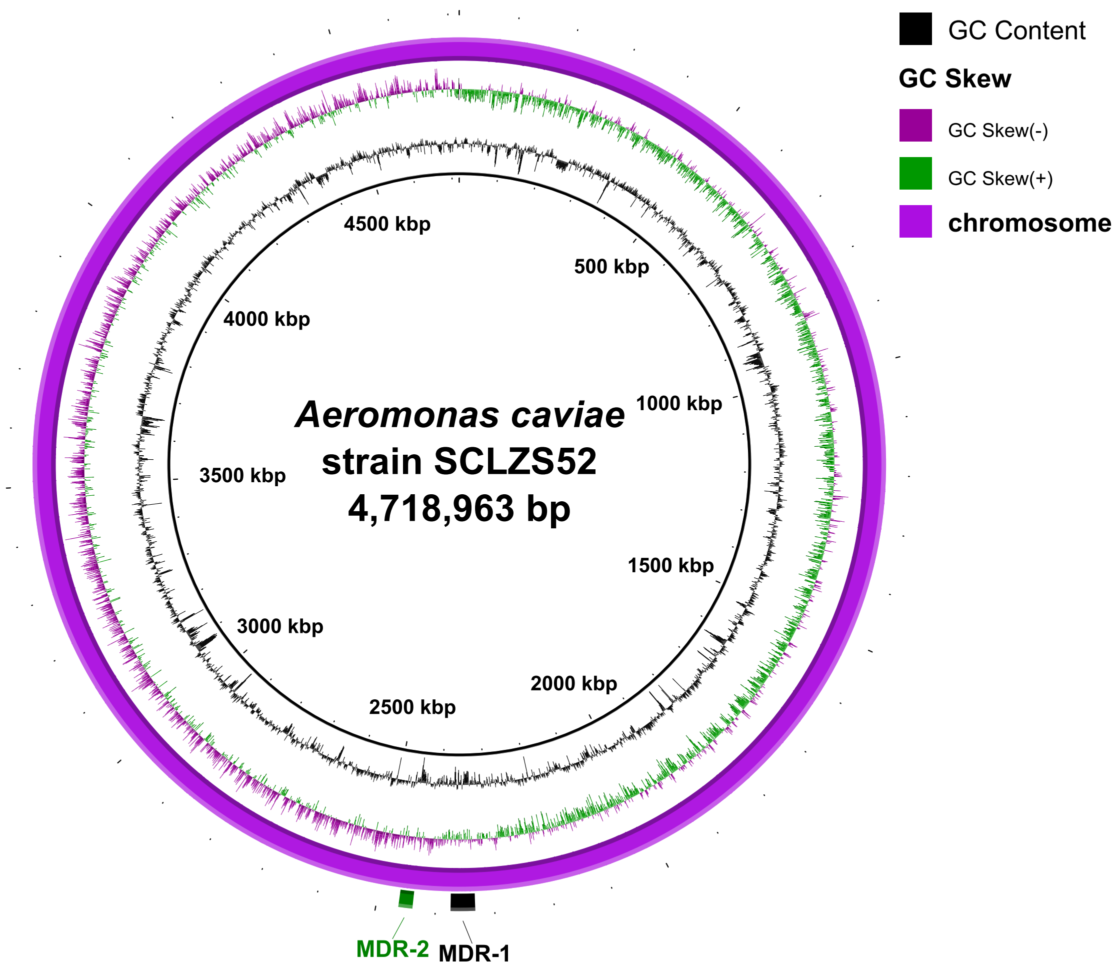

Figure S1 Schematic circular representation of the chromosome of *A. caviae* SCLZS52. The MDR-1 and MDR-2 regions are indicated by rectangles in black and green, respectively, at the outer circle.

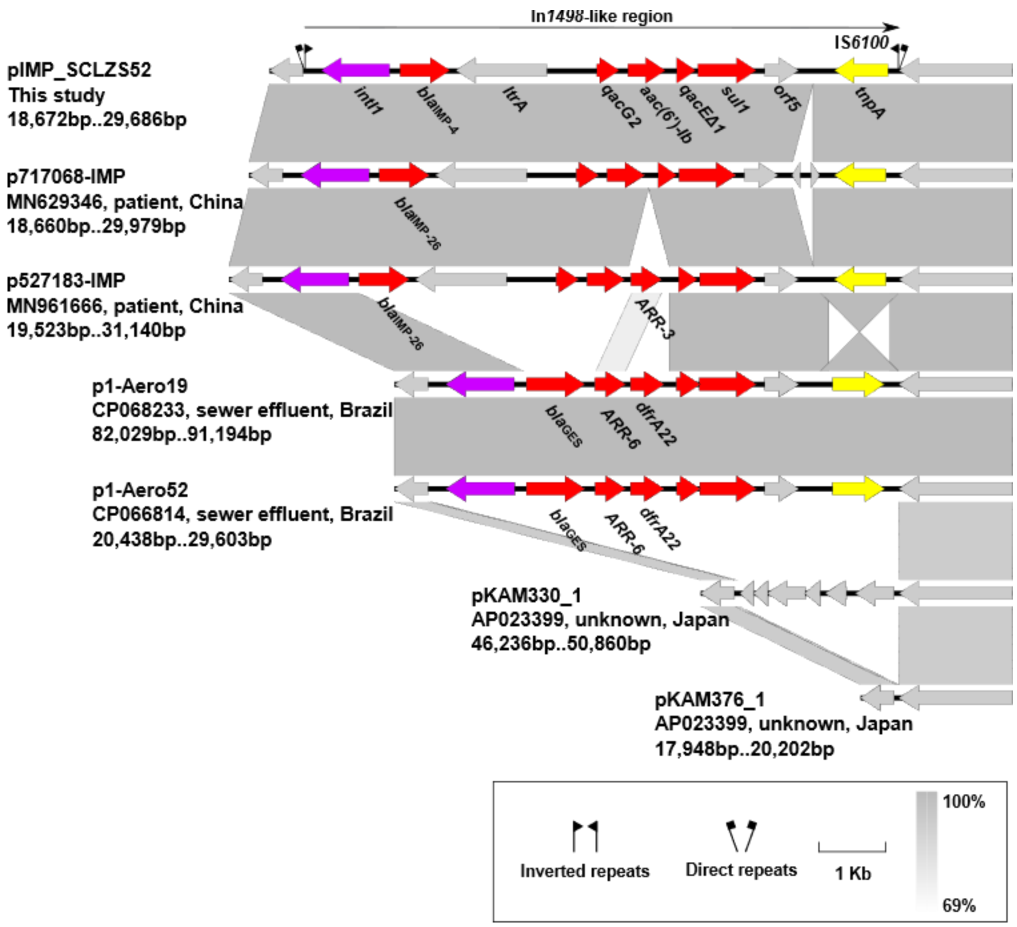

Figure S2 Comparison of the accessory modules of pIMP_SCLZS52-like plasmids. Resistance genes, mobile genetic elements and *intI1* are indicated in red, yellow, and purple, respectively. Gray shading indicates >69% nucleotide identity.
